# Protocol for mapping insertion sites of Tol2 transgenes in zebrafish using TransTag

**DOI:** 10.1016/j.xpro.2025.104259

**Published:** 2025-12-05

**Authors:** Xiaolu Wei, Vijaykumar Naidu, Paige Schneider, Patrick J. Murphy, Fanju W. Meng

**Affiliations:** 1 Department of Biological Sciences, Advanced Environmental Research Institute, University of North Texas, 1155 Union Circle #305220, Denton, TX 76203, USA; 2 Department of Biomedical Genetics, University of Rochester Medical Center, 601 Elmwood Avenue, Rochester, NY 14642, USA; 3 Department of Molecular Biology and Genetics, Cornell University, Ithaca, NY 14850, USA

**Keywords:** developmental biology, genetics, genomics, sequencing, model organisms, molecular biology

## Abstract

A valuable and widely utilized approach to study gene regulatory networks is through transgenic lines. However, detailed information of transgene insertion sites is often not available, complicating the interpretation of experimental results involving transgenes. Here, we present a protocol for mapping the insertion sites of zebrafish Tol2 transgenes, using the recently developed tagmentation-based TransTag technique. We describe detailed steps for preparing sequencing libraries and analyzing raw reads. This protocol can be used to robustly identify transgene insertion sites in zebrafish.

For complete details on the use and execution of this protocol, please refer to Meng et al.^1^

## Highlights

• Steps for mapping Tol2 transgene insertion sites using TransTag
• Instructions for preparing TransTag sequencing libraries
• Guidance on data analysis with step-by-step tutorials

## Abstract (editor highlights)

Publisher’s note: Undertaking any experimental protocol requires adherence to local institutional guidelines for laboratory safety and ethics.

## Graphical abstract

**Figure:**
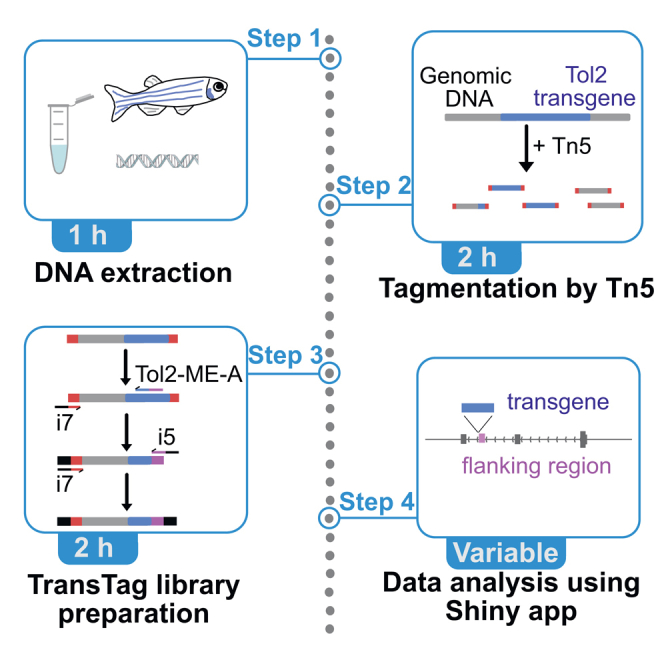

## Before you begin

Tol2 transposase mediated transgene insertion is one of the most popular transgenesis methods widely used among zebrafish research laboratories.^2^^,^^3^^,^^4^^,^^5^ However, precise insertion sites of most Tol2 transgenes are unknown, leading to potential issues of transgene expression being subject to positional effects, off-target integration and variations in transgene copy number.^6^^,^^7^^,^^8^^,^^9^ We recently developed a transgene mapping method, TransTag, for mapping Tol2 transgenes.^1^ Our TransTag method adapts Tn5 transposase-mediated tagmentation to generate genomic library, and can identify insertion sites from single or compound transgenic lines.^1^

**Figure 1:**
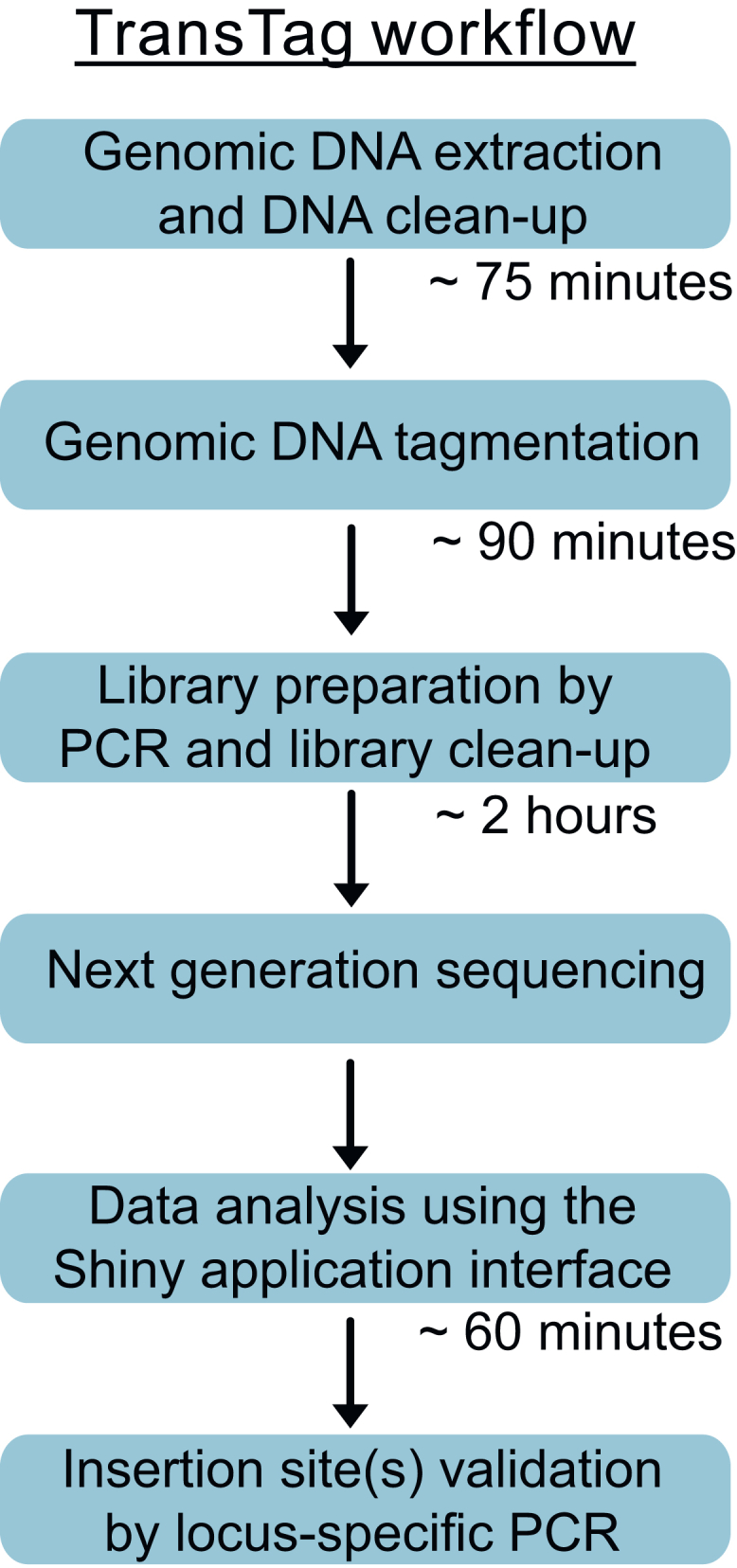
A diagram of TransTag workflow Figure reprinted and adapted with permissions from Meng et al., 2025.

This protocol provides detailed steps to generate TransTag libraries and analyze the raw sequencing reads through our Shiny app (Figure 1). It consists of two major sections. The first section details how to generate the TransTag sequencing library using genomic DNA isolated from zebrafish transgenic lines. The second section describes how to analyze the sequencing reads to identify transgene insertion sites.

This protocol is demonstrated for transgene mapping in zebrafish, however, TransTag can be used for other organisms where Tol2-mediated transgenesis method has been applied to. It can also be used to map insertion sites of transgenes generated by the piggyBac transposase. When applying our method to piggyBac transgenes, the primer will need to be designed specifically for the piggyBac transgenesis vector where inverted terminal repeats will be targeted,^10^^,^^11^ in a similar manner to Tol2 vector.^1^

**CRITICAL:** All steps in this protocol should be carried out under DNase-free conditions.

### Innovation

This protocol provides an efficient and robust method for identifying insertion sites of Tol2 transgenes. Compared to existing methods for transgene mapping, our method is simple to perform by adapting Tn5 transposase for sequencing library generation. Our method is suitable for sample multiplexing, allowing users to pool and sequence multiple samples simultaneously to further reduce sequencing cost. By providing an open-source R Shiny application for automatic data analysis, our method does not require bioinformatic expertise to analyze the data, thereby greatly facilitating transgene mapping for a broader research community.

### Institutional permissions

This study was approved by the Institutional Animal Care and Use Committee (IACUC) at the University of North Texas.

**Figure 2:**
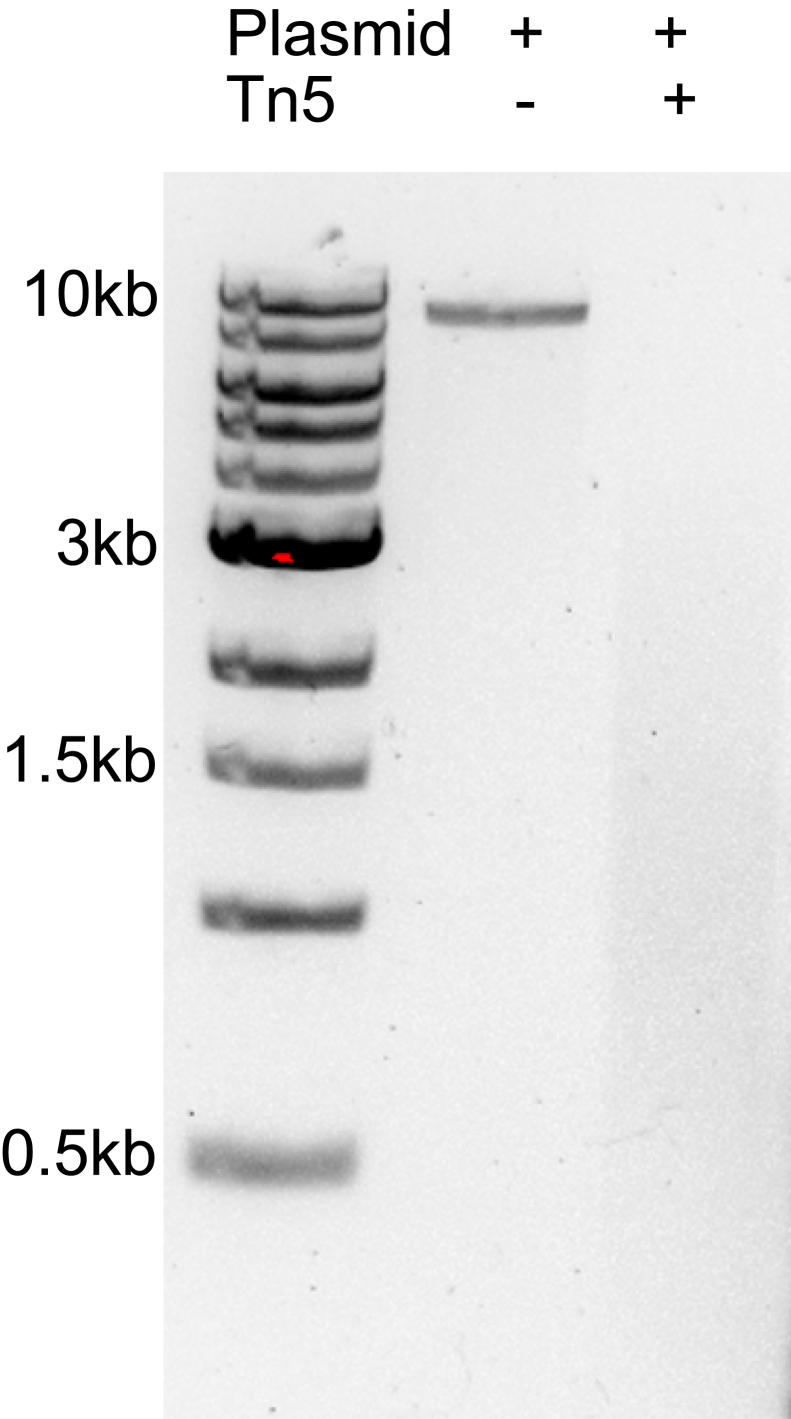
An agarose gel image demonstrating proper Tn5 activity using a linearized plasmid DNA

### Adapter/primer preparation

**Timing: 1 h**

1. Resuspend the mosaic end (ME) adapters B and Reverse oligonucleotides to 100 μM each in double-distilled water (ddH_2_O), and store them in −20°C.

***Note:*** The ME adapters B and Reverse oligonucleotides listed in the key resources table can be ordered directly from Integrated DNA Technologies (IDT).

2. Dilute i5 and i7 primers to 100 μM (stock concentration) each in ddH_2_O, and make a 10 μM (working concentration) aliquot and store them in −20°C.

***Note:*** The uniquely barcoded i5 and i7 primers used for genomic library preparation are listed in the key resources table and can be ordered from IDT. A series of i5 and i7 primers can be ordered depending on the need of sample multiplexing in a single sequencing run.

### Tn5 loading with ME adapter B

**Timing: 2 h**

3. In a PCR tube, mix 10 μL of each 100 μM ME Adapter B and Adapter Reverse. Mix by gently pipetting up and down for 10 times.
4. Incubate the oligo mixture for 3 min in a 95°C thermal cycler with heated lid (100°C).
5. After the incubation, allow the mixed oligos to cool down gradually to room temperature (20°C–25°C) in the thermal cycler without opening the lid (∼30 min).
6. To assemble Tn5 transposase, add 10 μL of 37 μM unloaded Tn5 protein to the PCR tube containing the mixed oligos. Pipet to mix and incubate at room temperature (20°C–25°C) for 1 h on a rotator.

***Note:*** Unloaded Tn5 transposase is commercially available via Diagenode (Catalog #: C01070010-10). Tn5 protein should be stored at −20°C and is stable for at least 6 months. Alternatively, Tn5 protein can be purified with the pTXB1-Tn5 plasmid (Addgene, #60240) using the published protocol.^12^

7. The assembled Tn5 can be aliquoted and stored at −20°C until use, and should be stable for at least 6 months.

### Test activity of loaded Tn5 transposase

**Timing: 3 h**

8. Set up two tagmentation reactions in PCR tubes on ice.
  a. Use 25 μL of 2× Tagmentation buffer and 100 ng of linearized plasmid DNA.
  b. Adjust total final volume to 50 μL using ddH_2_O.
  c. In the test tube, add 1 μL assembled Tn5 while omitting Tn5 in the negative control tube.
  d. Mix by gently pipetting up and down for 10 times.

***Note:*** Source of plasmid DNA is not critical, but purity and concentration should be high enough (e.g., plasmid size > 3 kb, concentration > 5 ng/μL) for visualization using standard agarose electrophoresis methods.

9. In a thermal cycler, incubate the PCR tube for 30 min at 37°C.
10. Add 1 μL of 10% SDS to the PCR tube to stop tagmentation. Pipet to mix and incubate for 5 min at room temperature (20°C–25°C).
11. Purify DNA using any DNA column purification kit. Run purified DNA on a 1% agarose gel to confirm that the plasmid DNA is successfully tagmented (Figure 2).

**CRITICAL:** Ensuring Tn5 activity is essential for generating the TransTag sequencing library. New batch of assembled Tn5 should be tested before performing TransTag library.

## Key resources table

**Table:** 

REAGENT or RESOURCE	SOURCE	IDENTIFIER
**Chemicals, peptides, and recombinant proteins**		

Unloaded Tn5 transposase	Diagenode	C01070010-10
Sodium hydroxide (NaOH)	Fisher Scientific	S318-1
Tris (base)	Fisher Scientific	4109–01
Magnesium chloride (MgCl_2_)	VWR	BDH9244-500G
N,N-Dimethylformamide	VWR	MK492904
Tween 20	VWR	97063–872
Sodium dodecyl sulfate (SDS)	VWR	IB07060
Ethanol	Fisher Scientific	BP2818500

**Critical commercial assays**		

DNA Clean & Concentrator Kit	Zymo Research	D4064
AMPure XP Reagent	Beckman Coulter	A63880
NEBNext High Fidelity 2× PCR Master Mix	New England Biolabs	M0541S

**Deposited data**		

TransTag	Meng et al.^1^	GEO: GSE281966

**Oligonucleotides**		

Mosaic End (ME) Adapter B GTCTCGTGGGCTCGGAGATGTGTATAAGAGACAG	Integrated DNA Technologies (IDT)	Picelli et al.^12^
ME Adapter Reverse [PHO]CTGTCTCTTATACACATCT	IDT	Picelli et al.^12^
ME-A-Tol2 primer TCGTCGGCAGCGTCAGATGTGTATAAGA GACAGTGAGTAAAATTTTCCCTAAGTACTTGT	IDT	Meng et al.^1^
I5 primer (10 bp barcode underlined) AATGATACGGCGACCACCGAGATCT ACACTCGTGGAGCGTCGTCGGCAGCGTC	IDT	Picelli et al.^12^
I7 primer (10 bp barcode underlined) CAAGCAGAAGACGGCATACGAGAT CGCTCAGTTCGTCTCGTGGGCTCGG	IDT	Picelli et al.^12^
Tol2_genotyping TGAGTAAAATTTTCCCTA AGTACTTGT	IDT	Meng et al.^1^
Medaka_*tyrosinase* TAGGAATGGAGACTACCTCCTG	IDT	Meng et al.^1^

**Recombinant DNA**		

pTBX1-Tn5	Addgene #60240	Picelli et al.^12^

**Software and algorithms**		

TransTag alignment free Shiny app https://menglab.shinyapps.io/transtag_alignmentfree/	Meng et al.^1^	https://github.com/fanjumeng/TransTag and https://doi.org/10.5281/zenodo.15557297
RStudio	Posit team.	https://posit.co/download/rstudio-desktop/

**Other**		

Step-by-step tutorial for TransTag library analysis using R Shiny app	Meng et al.^1^	https://doi.org/10.5281/zenodo.15557297
Thermal Cycler	Bio-Rad	1861096
NanoDrop spectrophotometer	Thermo Fisher Scientific	ND-ONEC-W
DynaMag PCR Magnet	Thermo Fisher Scientific	492025
Qubit 4 Fluorometer	Thermo Fisher Scientific	Q33238

## Materials and equipment

Recipes for the buffers used in this protocol are described below. Ensure that all the buffers are made with DNase-free water.

• **Alkaline lysis buffer**: 25 mM NaOH, pH=12.

Store the buffer at 20°C–25°C and use within 6 months.

• **Neutralization buffer**: 100 mM Tris-HCl, pH=8.

Store the buffer at 20°C–25°C and use within 6 months.

• **10% SDS**.

Store the buffer at 20°C–25°C and use within 6 months.

• **80% ethanol**: freshly made.
• **2× Tagmentation buffer** (100 μL):

**Table:** 

Reagent	Final concentration	Amount
1 M Tris-HCl, pH 7.5	20 mM	2 μL
1 M MgCl_2_	10 mM	1 μL
N,N-Dimethylformamide	20%	20 μL
1×PBS	N/A	66 μL
10% Tween 20	0.2%	2 μL
ddH_2_O	N/A	9 μL
Total	N/A	100 μL

Store the buffer at 20°C–25°C and use it on the same day of performing tagmentation.

## Step-by-step method details

### Genomic DNA extraction and cleanup

**Timing: 75 min**

These steps describe the procedures of extracting and cleaning up genomic DNA from zebrafish tissues.

1. Genomic DNA extraction.
  a. Transfer the zebrafish tissue to a clean PCR tube.
  b. Add 50 μL Alkaline lysis buffer directly to the PCR tube. In a thermal cycler, incubate the PCR tube for 30 min at 95°C.
    **CRITICAL:** Make sure the zebrafish tissue is completely submerged in the alkaline lysis buffer to ensure proper DNA extraction.
  c. After the incubation, vortex the tube on a vortex mixer for 3–5 s. Add 50 μL Neutralization buffer to the tube and pipet to mix.
  d. Spin down for 3–5 s on a bench-top microcentrifuge, and transfer supernatant to a new tube to keep the genomic DNA.
    ***Note:*** Genomic DNA can be extracted from adult zebrafish fin clip, whole embryo or larvae. Other DNA extraction methods can also be used, e.g. phenol-chloroform DNA extraction.
2. DNA clean-up.
  a. Clean up genomic DNAs using a Zymo Research DNA Clean & Concentrator kit or other similar kits according to manufacturer’s standard protocol.
  b. Elute DNA in 25 μL ddH_2_O, and quantify DNA concentration using a NanoDrop spectrophotometer.

***Note:*** (optional) Ensure initially purified DNA is of high quality and not degraded via standard agarose-based electrophoresis methods.

**Pause point:** Genomic DNA can be stored at −20°C for several weeks prior to performing genomic DNA tagmentation and TransTag library preparation.

### Genomic DNA tagmentation using loaded Tn5

**Timing: 90 min**

These steps describe methods of performing genomic DNA tagmentation and purifying these DNA fragments.

3. Set up a 50 μL tagmentation reaction in a PCR tube on ice. Pipet gently to mix the sample.

**Table:** 

Reagent	Amount
2× Tagmentation buffer	25 μL
Genomic DNA (100 ng)	Variable
ddH_2_O	Adjust total final volume to 50 μL
Assembled Tn5	1 μL

***Note:*** 100 ng genomic DNA is sufficient for generating a TransTag library.

4. In a thermal cycler, incubate the PCR tube for 30 min at 37°C.
5. After the incubation, add 1 μL of 10% SDS to the PCR tube. Pipet gently to mix the sample and incubate the tube for 5 min at room temperature (20°C–25°C).
6. Perform DNA clean-up using AMPure XP beads.
  a. Shake to resuspend the AMPure beads before using it.
  b. Add 40 μL (0.8× volume of sample) of AMPure XP beads to the PCR tube. Mix thoroughly by pipetting up and down for at least 10 times.
  c. Let the tube incubate for 5 min at room temperature (20°C–25°C).
  d. Place the tube on a DynaMag PCR magnet and allow to clear. Withdraw liquid carefully without disturbing the beads.
  e. Add 200 μL of freshly made 80% ethanol to the tube and incubate for 30 s at room temperature (20°C–25°C). Withdraw liquid without disturbing the beads.
  f. Repeat step e for a total of two washes with 80% ethanol.
  g. After a quick spin of the tube, completely remove the remaining 80% ethanol with a 20 μL pipette.
  h. Keep the tube on magnet stand and air-dry the beads for 5 min.
  i. Remove the tube from magnet stand, and then add 25 μL of ddH2O and vortex on full for 30 s. Incubate the tube for 5 min at room temperature (20°C–25°C).
  j. Place the tube back to magnet stand for 1 minute. Transfer liquid to a new PCR tube.

***Note:*** Do not allow beads to completely dry while changing buffers, as this could compromise DNA quality or yield.

**CRITICAL:** Tn5 transposase activity depends on Mg^2+^, so it is critical to avoid any chelators (e.g. EDTA, EGTA) in reaction buffers.

### TransTag library preparation in two steps

**Timing: 2 h**

These steps generate TransTag library from tagmented genomic DNAs using two steps of PCR reactions.

7. Set up the first round of PCR on ice using the ME-A-Tol2 primer and an i7 primer.

**Figure 3:**
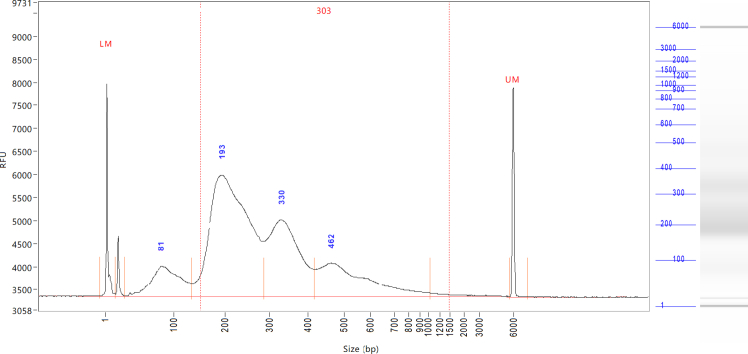
Fragment analysis of a TransTag library

**Table:** PCR reaction: first round

Reagent	Amount
NEBNext 2× HiFi Master Mix	12.5 μL
Tagmented DNA	10.5 μL
i7 primer (10 μM)	1 μL
ME-A-Tol2 primer (10 μM)	1 μL

**Table:** PCR condition: first round

Steps	Temperature	Time	Cycles
Gap filling	72°C	5 min	1
Initial Denaturation	98°C	30 sec	1
Denaturation	98°C	10 sec	20 cycles
Extension/Elongation	63°C	15 sec	
Final extension	72°C	60 sec	1
Hold	4°C

***Note:*** If you plan to pool multiple TransTag libraries into one sequencing run, you can use a uniquely barcoded i7 primer for each of your samples. Keep a good note of primers used as it is critical for proper sample demultiplexing after sequencing.

8. Set up the second round of PCR using the i5 and i7 primers.

**Table:** PCR reaction: second round

Reagent	Amount
NEBNext 2× HiFi Master Mix	25 μL
First round PCR product	12.5 μL
i5 primer (10 μM)	2 μL
i7 primer (10 μM)	2 μL
ddH_2_O	8.5 μL

**Table:** PCR condition: second round

Steps	Temperature	Time	Cycles
Initial Denaturation	98°C	30 sec	1
Denaturation	98°C	10 sec	20 cycles
Extension/Elongation	63°C	15 sec	
Final extension	72°C	60 sec	1
Hold	4°C

***Note:*** In the second round of PCR, each TransTag library is generated using a unique i5 and i7 primer combination. Keep a good note of primers used as it is critical for proper sample demultiplexing after sequencing.

**CRITICAL:** Use the exact same i7 primer during both first and second rounds of PCR reactions for the same sample.

9. Post-PCR clean-up of TransTag library using AMPure XP beads.
  a. Clean up TransTag library using 55 μL (1.1× volumes of sample) of AMPure beads to each PCR sample, and elute in 15 μL ddH2O as the final library. The detailed procedures are the same as described in step 6.
  b. Transfer liquid to a new PCR tube with a pipette and quantify DNA concentration using Qubit.

***Note:*** This is the final TransTag library that is ready for sample multiplexing, quality control by Bioanalyzer or TapeStation (Figure 3), and DNA sequencing. TransTag library can be stored at −20°C.

***Note:*** The concentration of a TransTag library can be measured by Qubit. Typical concentration of a final library is about 20 ng/μL. Uniquely barcoded TransTag libraries can be multiplexed for sequencing, either on its own or as spike-in samples for other types of genomic libraries.

**CRITICAL:** We recommend using 150 bp sequencing platforms as the longer reads increase the chance of precisely mapping transgene insertion sites. Sequencing depth of 1–5 million reads per sample is often sufficient to identify transgene insertion sites.

### Alignment-free analysis of TransTag data using a Shiny app

**Timing: Variable**

These steps instruct users to analyze TransTag sequencing data using the online Shiny application.

10. We provide a R Shiny application in a web browser. This app allows automatic analysis of the TransTag sequencing reads without alignment: https://menglab.shinyapps.io/transtag_alignmentfree/.
11. Upload the raw sequencing reads from the TransTag library for processing (first read R1 file for paired end reads).

***Note:*** An example input file is Sample1_R1.fastq.gz. The shinyapps.io can only process input files smaller than 1 GB. If input file size is larger than 1 GB, users can download and run TransTag_alignmentFree.ShinyApp.R on their own computers to launch the same Shiny app interface. Links to the R Shiny code and further instructions is https://github.com/fanjumeng/TransTag.

**Figure 4:**
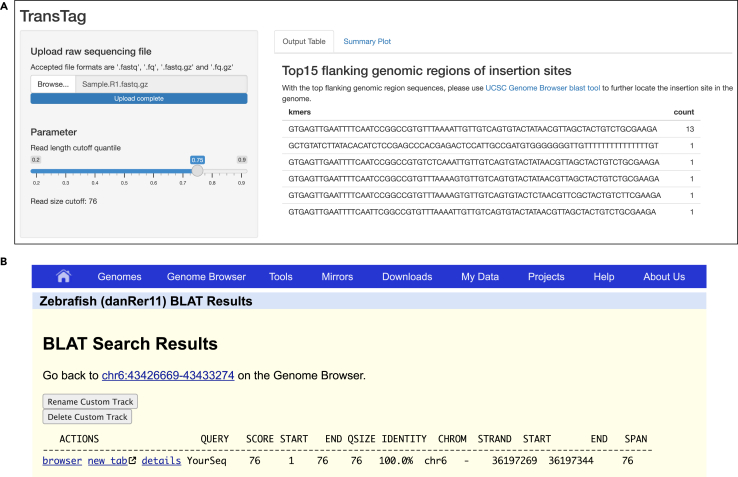
Example Shiny app output and identification of transgene insertion site (A) Shiny app displays top15 identified genomic regions flanking transgene insertion sites directly from raw sequencing reads. (B) Identification of transgene insertion site using the BLAT tool of UCSC genome browser.

12. Slide through the read length cutoff quantile values (between 0.2 to 0.9) to change the cutoff values for flanking genomic regions. Examine the top 15 most frequent genomic regions and their count numbers (Figure 4A).

***Note:*** The default value for read length cutoff quantile is set at 0.75. The displayed ranking is based on numbers of reads containing the flanking genomic regions.

13. Blast the flanking genomic regions to zebrafish genome to identify the genomic coordinates of transgene insertion sites (Figure 4B).

**CRITICAL:** It is essential to perform validation experiments to confirm the transgene insertion site. Locus-specific PCR primers should be designed and used for PCR validation.

## Expected outcomes

This protocol describes the steps to generate the TransTag library for identifying Tol2 transgene insertion sites in zebrafish. The procedure involves assembling Tn5 transposase with adapters, applying the assembled Tn5 for genomic DNA tagmentation, and generating the TransTag library. To facilitate downstream data analysis, a custom Shiny application is provided, allowing users to analyze TransTag sequencing data without requiring bioinformatics expertise.

Successfully Tn5 loading can be confirmed by plasmid DNA fragmentation. During the Tn5 activity testing step, linearized DNA is efficiently fragmented by Tn5, yielding the expected pattern on an agarose gel (Figure 2). Genomic DNA can then be used to generate the TransTag library, with a representative DNA fragment size distribution shown in Figure 3. Sequencing reads can be uploaded directly into the Shiny app, which provides automated analyses and visualizations. An example output is presented in Figure 4. Based on the sequence of flanking genomic DNAs, the insertion site of transgenes can be identified (Figure 4B).

Altogether, this protocol provides a straightforward and robust method for mapping transgene insertion sites.

## Limitations

Our TransTag method can robustly identify insertion sites of Tol2 transgenes from a low amount (e.g., 1 - 5 million reads) of sequencing reads.^1^ However, based on our current protocol, we found that many sequencing reads are not chimeric reads. It is possible that these reads are generated due to non-specific priming events during PCR. Further optimization of the sequence of primer would address this limitation and improve the efficiency of our TransTag method.

As shown by prior studies and our recent study,^1^^,^^13^ Tol2-independent transgene insertions are relatively frequent. Under this condition, transgenes often retain part of the transgenesis vector sequences which exceeds the length limit that TransTag can identify based on current sequencing platforms (150 bp length of reads). Therefore, our TransTag method is not able to map insertion sites of such transgenes.

## Troubleshooting

### Problem 1

Loaded Tn5 is not active for tagmentation (step 11 of test activity of loaded Tn5 transposase).

### Potential solution

• Double check the steps of Tn5 loading.
• Confirm that tagmentation buffer is prepared correctly. Make sure Mg^2+^ is added and no chelators in the buffer.

### Problem 2

Low concentration of extracted genomic DNA (step 2).

### Potential solution

• Make sure the collected zebrafish tissues (e.g., fin clip, embryos) are completely submerged in the alkaline lysis buffer during the genomic DNA extraction step.

### Problem 3

Low concentration of a TransTag library (step 9).

### Potential solution

• Confirm Tn5 activity is normal during the Tn5 activity testing step.
• Confirm that tagmentation buffer is prepared correctly. Make sure Mg^2+^ is added in the buffer, and no chelators in reaction buffers.
• Make sure the same i7 primer is used for the same sample during step 7 and step 8.
• Make sure the AMpure XP beads cleanup step is handled properly.

### Problem 4

Failure in uploading the raw sequencing data to the online Shiny app or to Shiny app in R from a local computer (step 11).

### Potential solution

• If the following error “Disconnected from the server. Reload” occurs with the online Shiny app, it is because the input file exceeds the size limit (1 Gb). As an alternative, you can download the R Shiny code and open it in your local computer to analyze the sequencing reads.
• If “Maximum upload size exceeded” error occurs, modify “options (shiny.maxRequestSize =)” in the TransTag_alignmentFree.ShinyApp.R script to increase the input file size limit. The default size limit is 20 GB.

### Problem 5

Failure to display flanking genomic regions using the Shiny app (step 12).

### Potential solution

• The failure could be due to low sequencing depth. If this is the case, increasing sequencing depth to 10–20 million reads could improve the possibility of identifying insertion sites.
• It is also possible that this transgene is generated via a Tol2-independ event which retains part of the transgenesis vector. In this case, watch out for warning message indicating potential Tol2-independent integration event(s).

### Problem 6

Failure to identify transgene insertion sites based on BLAST results of flanking genomic regions (step 13).

### Potential solution

• The expected result for a single transgene insertion site is that BLAST will return a single unique match in the genome using the flanking genomic region identified. However, if multiple hits were returned for the BLAST, it is possible that the transgene is inserted into some repetitive elements which often has many copies.
• Multiple insertions could occur during Tol2-mediated transgenesis process, leading to variations in transgene copy number. If several candidate loci are identified, PCR validation should be performed using locus-specific PCR primers to further identify the true transgene insertion site.

## Resource availability

### Lead contact

Further information and requests for resources and reagents should be directed to and will be fulfilled by the lead contact, Fanju W. Meng (fanju.meng@unt.edu).

### Technical contact

Technical questions on executing this protocol should be directed to and will be answered by the technical contact, Xiaolu Wei (xiaolu.wei@unt.edu).

### Materials availability

This study did not generate new unique reagents.

### Data and code availability

All primary sequencing data have been deposited in the NCBI Gene Expression Omnibus under accession code (GEO: GSE281966). The R Shiny code for TransTag data analysis is available at GitHub (https://github.com/fanjumeng/TransTag) and Zenodo (https://doi.org/10.5281/zenodo.15557297).

## Acknowledgments

We thank the University of Rochester Genomics Research Center for sequencing our TransTag library. This study was supported by the National Institutes of Health (R35GM137833 and R01HD105489 to P.J.M.) and start-up funds from the University of North Texas (F.W.M.).

## Author contributions

Conceptualization, P.J.M. and F.W.M.; formal analysis, X.W., V.N., and P.S.; writing – original draft, X.W. and F.W.M.; writing – review and editing, all authors; supervision, F.W.M.

## Declaration of interests

The authors declare no competing interests.
